# Monthly Report of Diseases

**Published:** 1800-04

**Authors:** 


*** *The following valuable Report, which will in future be regularly continued, was received this Month too late to appear in its proper place.*

### MONTHLY REPORT of DISEASES,

Admitted under the Care of the PHYSICIANS of the FINSBURY DISPENSARY, St. John's Square, Clerkenwell.

*The Diſtrict, in which the Patients of the Finsbury Diſpenſary are viſited, comprehends the Pariſhes of St. James, and of St. John, Clerkenwell; of St. Luke; of St. Saviour within and without; of St. Bartholomew, the Great and the Leſs; the Liberties of the Rolls, and of Glaſs-Houſe Yard; the Town of Iſlington; the Pariſhes of St. Pancras; of St. Andrew, Holborn; and of St. George the Martyr, Queen's-ſquare. This Tract of Ground may properly enough be termed, a North Weſtern Diſtrict of the Metropolis.*

LIST of DISEASES, &c. from Feb. 20, to March 20.

| | No. of Caſes. | | No. of Caſes. |
|---|---|---|---|
| Continued Fever - - | 15 | Hyſteria - - - - - - | 2 |
| Catarrhal Fever - - - | 3 | Hypochondriaſis - - - | 2 |
| Scarlet Fever - - - - | 7 | Cephalæa - - - - - | 4 |
| Aphthous Sore Throat - | 3 | Enterodynia - - - - - | 2 |
| Pneumonia - - - - - - | 3 | Diarrhœa - - - - - - | 11 |
| Peripneumonia Notha - - | 4 | Conſtipatio - - - - - | 2 |
| Eryſipelas - - - - - - | 1 | Inteſtinal Hæmorrhagy - | 2 |
| Hæmoptyſis - - - - - | 3 | Phyſconia Abdominalis - | 3 |
| Rheumatiſm - - - - - | 4 | Dropſy - - - - - - - | 6 |
| Dyſentery - - - - - - | 2 | Gout - - - - - - - | 2 |
| Pulmonary Complaints without Fever - - - - | 54 | Paraplegia - - - - - - | 1 |
| Dyſpepſia - - - - - - | 8 | Hemiplegia - - - - - | 1 |
| Aſthenia - - - - - - | 11 | Pulmonary Conſumption - | 5 |
| Chloroſis and Amenorrhœa | 7 | Urinæ Incontinentia - - | 1 |
| Menorrhagia - - - - - | 3 | Hooping Cough - - - | 3 |
| Leucorrhœa - - - - - - | 3 | Infantile Fever - - - | 4 |
| Nephralgia Calculoſa - - | 3 | Meſenteric Fever - - - | 2 |
| | | Chronic Cutaneous Diſeaſes | 9 |

The periodical account of diſeaſes, thus offered to the Public, is not intended as an exact epitome of the ſtate of epidemics, whether of the acute or chronic kind, which prevail throughout the whole of the metropolis. The different circumſtances of the rich and poor, occaſion a ſtriking diverſity in their diſeaſes: while, by cleanlineſs and a free circulation of air, by a generous diet, warm cloathing, and a dry and comfortable habitation, the one claſs eſcape the effects of febrile and other contagions, and neither feel the debility of want, nor the inclemency of winter; a plain and ſcanty meal, earned by the ſweat of the brow, a hardineſs of conſtitution, and a mind little agitated by care, exempt the other, though not in an equal degree from the diſorders which luxury, indolence, and mental anxiety entail on their opulent, and apparently more enviable, neighbours. A faſhionable phyſician attending on the rich, and another in the ſame diſtrict, and at the ſame time, viſiting the ſick poor, would preſent liſts of diſeaſes widely different: gout and hyſteria might ſtand foremoſt in the one; contagious fever, and dyſentery in the other. It is to be lamented, however, that from the ſedentary life to which a great portion of the poor in large cities are ſubjected, and from the univerſal and exceſſive uſe of tea, and particularly of ſpirituous liquors, which prevails amongſt them, a conſiderable number of complaints, which were once almoſt peculiar to the rich, are now ſuperadded to thoſe which more eſpecially attend a ſtate of poverty; ſo that the report of the diſeaſes of the lower claſs may, in too many inſtances, be regarded as a general ſpecimen.

It is well known, that an acute diſeaſe is often epidemic in one part of an extenſive city, while in another few or no traces of it are to be found; that frequently it does not, till after a conſiderable time, ſpread itſelf univerſally; or perhaps, after a partial extenſion only, it becomes extinct, leaving many parts wholly untouched by its influence.

It is obvious alſo, that of many kinds of epidemics, a ſmall proportion of caſes only fall under the notice of medical practitioners. This obſervation applies particularly to thoſe diſeaſes of the poor which are of ſhort duration, or are mild in their nature. Hence, in a public diſpenſary, it is found, that the caſes of meaſles, of ſcarlet fever, and of hooping-cough, bear not the ſame ratio to the actual prevalence of thoſe complaints, as the caſes of moſt other diſeaſes which occur in ſimilar practice. It may be noticed alſo, that an inſtance of ſmall pox is rarely to be met with at a diſpenſary, owing probably to the general uſe of inoculation, and to the eſtabliſhment of a ſmall-pox hoſpital.

Notwithſtanding theſe circumſtances, however, it is preſumed, from the example of ſimilar reports lately preſented to the public, that ſuch an one as that now propoſed to be given, may afford ſome uſeful information reſpecting many epidemic diſeaſes, both chronic and acute, ſuch for inſtance as of fevers, pneumonia, dyſentery, diarrhœa, rheumatiſm, catarrhal affections, &c.

Were a certain number of phyſicians, in different parts of the metropolis, as well thoſe who belong to medical charities, as thoſe who are engaged in extenſive private practice, to unite in publiſhing periodically the reſult of their obſervations; an accurate and comprehenſive view would then be regularly obtained of the ſtate and progreſs of all the diſeaſes which prevail throughout London; and there is good reaſon to believe, that, from ſuch a plan well conducted, a body of evidence might, in no long time, be produced, which would elucidate many obſcure and intricate points relating to epidemic diſeaſes. That it would, in the mean time, prove an uſeful guide to practitioners in general, no one who is acquainted with the influence of epidemic diſorders on each other will venture to deny.

The moſt important claſs of diſeaſes, enumerated in the foregoing liſt, is that of continued fevers. Under this term are comprehended the typhus and ſynochus, in their different degrees and varieties rieties, whether ariſing from contagion, or from cold and other debilitating cauſes. Fevers, we are happy to ſay, have, for ſome time paſt, been gradually declining, both as to the frequency of their occurrence, and the malignity of their ſymptoms. The number of caſes during the months of October, November, and December, of the laſt year, were to thoſe which have happened ſince the beginning of the preſent, nearly in the proportion of four to one.

The accurate deſcription of the fever which prevailed at the latter end of the autumn in another quarter of the town, (inſerted in the Medical Journal for November) accords in general with the ſymptoms of that which occurred within the ſame period in the Finſbury diſtrict; there were, however, ſome circumſtances of diſtinction. The latter was decidedly of a leſs malignant nature; for, of between fifty and ſixty caſes, which fell under the obſervation and treatment of one of the phyſicians, in October and November, three only terminated in death; whereas, in the former, the proportion of fatal caſes was as one in four,—ten out of forty-one having died. In the latter alſo, a diſtinct criſis was ſeldom or ever obſervable, the ſigns of amendment ſhewing themſelves in the moſt gradual manner, ſo that it was difficult to mark the exact time of their appearance. Theſe ſigns for the moſt part were, the patient becoming compoſed, and falling into an eaſy and refreſhing ſleep, after a ſtate of watchfulneſs and irriration; his expreſſing himſelf to be more comfortable in his general feelings; the eyes and countenance reſuming their natural aſpect; the edges of the tongue beginning to look clean; the pulſe becoming ſtronger, more ſteady; and leſs frequent; a diminution of the heat of the ſkin, and a return of its uſual ſoftneſs. Of theſe beneficial changes, ſometimes one, ſometimes another, gave the earlieſt notice of recovery; but the firſt ray of hope was generally reflected from the eyes, and the features of the face.

The moſt common period of the termination of this fever was the end of the ſecond week, or about the fourteenth or fifteenth day. In a few caſes, the diſeaſe was protracted to an unuſual length, in one of which, an univerſal yellowneſs of the ſkin, pain about the region of the liver, and violent vomiting, twice occurred; at firſt during the third week, and the ſecond time at the end of the ſixth, when it proved fatal. The patient, who was a woman rather advanced in life, there was reaſon to believe, had been addicted to habits which particularly injure the hepatic ſyſtem. In ſeveral, the diſeaſe terminated within the firſt week: in the greater part of theſe, there had been an opportunity of adminiſtering an emetic ſoon after its commencement. The decided power of emetics, in cutting ſhort the fever, or in rendering it more mild in its ſymptoms, was ſtrikingly exemplified in a variety of inſtances. A ſtate of watchfulneſs and irritation was more than uſually common, and proved exceedingly diſtreſſing. During the firſt ſtage of the fever, it was ſeldom removed, and often aggravated, by remedies, particularly by opium. This medicine, however, about the middle or end of the ſecond week, was employed with the very beſt effects, eſpe-

 cially cially when given in doſes not exceeding a quarter of a grain, repeated at intervals of about five or ſix hours. In one caſe, the *tinct. opii*, with a ſuitable quantity of *linim. ſaponis*, was rubbed on the legs and thighs, and was ſucceeded by profound ſleep, after it had been adminiſtered internally, without any ſoporific operation whatever. In ſome patients there were excruciating pains of the limbs, ſo that they cried out as though they had been affected with acute rheumatiſm. In ſome alſo, eſpecially about the beginning of November, there were conſiderable aphthous ulcerations in the throat. A cough was a moſt common attendant, and not unfrequently was ſo violent and harraſſing as to require particular attention. In a few, pneumonic inflammation ſupervened, forming a combination of ſymptoms, than which there are few more embarraſſing to the phyſician, in the hiſtory of acute diſeaſes. To abate the inflammation in theſe circumſtances, it is ſeldom that more powerful means are admiſſible, than the application of leeches and bliſters about the thorax. The cautious uſe of antimonials and opiates may be joined to that of demulcents and diluents, and a gentle emetic may ſometimes be had recourſe to with advantage, if there be not too great debility. Perhaps calomel, in alterative doſes, joined with opium, as recommended by Drs. Hamilton, Duncan, and Wright, is here eſpecially indicated. In one caſe, which ended favourably, it was tried to the amount of about 5 grains in forty-eight hours, joined with a ſmall quantity of opium and antimonial powder. What was its preciſe effect, or whether it had any effect at all in removing the inflammation, could not be determined from a ſingle inſtance, when other means, as bliſters and an emetic, had alſo been employed.

The bark, although it did not appear poſitively hurtful, was certainly attended with no advantage in the early ſtage of the diſeaſe; in the latter periods, however, it was manifeſtly uſeful in ſupporting the ſtrength, and apparently in accelerating the extinction of the fever.

The waſhing the body with cold water was tried on a few patients, in ſome of whom it ſeemed to bring on ſevere catarrhal ſymptoms, and it was very unpleaſant to the feelings of others; it was preſcribed only when there was a preternatural degree of heat, an increaſed action of the arterial ſyſtem; circumſtances pointed out by Dr. Currie as demanding and rendering ſafe its adminiſtration. Bliſters were not employed, except for the relief of topical affections.

The fever, as it has prevailed during the preſent month, has aſſumed the character of the typhus mitior.

It is not wonderful, that, of all diſeaſes, thoſe affecting the organs of reſpiration ſhould ſtill continue the moſt numerous, when it is conſidered how much they are influenced by the ſenſible qualities of the atmoſphere, which for a long time paſt have had a peculiar tendency to produce them.

Of the caſes of ſcarlatina, noticed in the liſt, four were ſucceeded by anaſarca, which yielded with difficulty to the remedies employed. The patients were children.

A diarrhoea has lately been very common, and has ſupervened on many other diſorders, both chronic and acute.

The caſe of paraplegia occurred in a delicate girl, between thirteen and fourteen years of age, without any obvious cauſe. It took place in the night during ſleep; on awaking from which, ſhe found herſelf totally deprived of the power of motion; with ſome diminution of ſenſation in the lower extremities. At the expiration of a fortnight, on awaking again from ſleep, ſhe was agreeably ſurpriſed at being able to get out of bed, and walk about the room. She has been ſimilarly affected three or four times within the laſt two years. During the complaint ſhe is low-ſpirited, and loſes her appetite; her bowels alſo are remarkably torpid.

W. W.
J. R.

---

NEW MEDICAL PUBLICATIONS IN MARCH.

